# Clinical Features of Anthroponotic Cutaneous Leishmaniasis in a Major Focus, Southeastern Iran, 1994–2014

**Published:** 2017

**Authors:** Ahmad KHOSRAVI, Iraj SHARIFI, Alireza FEKRI, Alireza KERMANIZADEH, Mehdi BAMOROVAT, Mahshid MOSTAFAVI, Mohammad Reza AFLATOONIAN, Alireza KEYHANI

**Affiliations:** 1. Leishmaniasis Research Center, Kerman University of Medical Sciences, Kerman, Iran; 2. Research Center of Tropical and Infectious Diseases, Kerman University of Medical Sciences, Kerman, Iran

**Keywords:** Clinical features, Cutaneous leismaniasis, *Leishmania tropica*, Iran

## Abstract

**Background:**

Cutaneous leishmaniasis (CL) is associated with a broad and complex clinical spectrum of diseases. The objectives of this study were to assess the clinical features and identification of the causative agents of CL in a well-known focus of anthroponotic CL (ACL) caused by *Leishmania tropica*, southeast Iran.

**Methods:**

This study was performed randomly as a descriptive cross-sectional survey to evaluate 2000 CL patients by active and passive case-detection approaches in Kerman Province from 1994 to 2014. The ACL patients were confirmed by direct smear and 600 cases by one or a combination of intrinsic methods.

**Results:**

Children aged <10 yr old were the most infected patients (*P*<0.001). The majority of the CL lesions were located in hands (46.3%), face (34.1%), legs (14.3%), and other parts of the body (5.3%). The mean number of lesions was 1.5 and most of the patients had single lesion (65%).Typical clinical lesions included papule (36.8%), followed by ulcerated nodule (20.7%), plaque (18.4%), and ulcerated plaque (18.5%). While among atypical clinical features, leishmaniasis recidivans (LR) (4.7%) and leishmanid (0.3%) were the dominant forms, followed by diffuse, disseminated, sporotrichoid, and erysipeloid types, 0.1% each, and then lymphedematous, lymphadenic, hyperkeratotic, paronychial, and mutilating types, 0.05% each. Based on various intrinsic methods the parasites isolated from the lesions were characterized as *L. tropica.*

**Conclusion:**

ACL due to *L. tropica* presents numerous cases of localized form and diverse uncommon clinical presentations, which mimic other disease conditions. Therefore, physicians should be aware of such manifestations for selecting appropriate treatment modality.

## Introduction

Leishmaniasis represents a major vector-borne disease with significant morbidity and mortality, reported from 98 countries and territories (1) in Indian sub-continent, Africa, Americas, and the Eastern Mediterranean Region. Globally, there are 1.5–2 million new cases and 350 million people are at the risk of infections with the overall prevalence rate of 12 million cases (2). Cutaneous leishmaniasis (CL) is the most prevalent form that encompasses 75% of the total cases (3).

Anthroponotic CL (ACL) and zoonotic CL (ZCL) due to *L. tropica* and *L. major,* respectively are present in 18 out of 31 provinces (4) with different clinical features and diverse epidemiological characteristics (5). The clinical manifestations of different types of CL have been classified in various ways. Most authors have traditionally divided ACL and ZCL into two distinct forms of “wet” or rural type and “dry” or urban type, respectively, without considering the pathogenesis (6). CL evolves in four major clinical presentations. Acute or localized CL represents the most typical clinical form of the disease in the Old World (7). The initial lesion starts as an erythematous papule and then develops to a larger and painless nodule. The nodule may progressively ulcerate over a period of two weeks to six months to a volcanic form (8, 9). Spontaneous healing eventually results in a depressed cutaneous scar and long-lasting protection from the disease (10).

Acute CL may evolve to one of the several clinical forms of the disease depending on the complexity of the host immune responses, parasite species, and diverse biological interactions between the causative agent and reservoir host (8, 11–13). ACL infection lasting for over two years should be considered chronic CL (CCL), more frequently observed by *L. tropica* infection (8). A well-defined case of CCL is leishmaniasis recidivans (LR). LR or lupoid leishmaniasis is predominantly caused by *L. tropica* in ACL foci (14) and LR is characterized by the development of recrudescing papules or nodules formed around or in the site of primary healed lesions. Diffuse CL (DCL) is basically presented by *L. aethiopica* in the Old World (8) and *L. mexicana* complex in the New World (15). The initial nodules do not ulcerate and new progressing lesions often develop on the face or on the whole body, which closely resembles lepromatous leprosy (8).

Clinical evaluation of CL lesions along with the precise identification of its causative agent is essential for selecting appropriate treatment modality and strategy planning, particularly in ACL areas where control approaches are restricted to early detection and prompt treatment of patients (7). Since CL mimics other conditions, particularly microbial and fungal infections, knowledge of the disease is necessary for any dermatologic practice in endemic countries.

Therefore, the present study aimed to assess various aspects of clinical features of ACL in the major endemic areas of Kerman Province, southeast Iran. This work is the first large-scale project in ACL endemic areas which presents various clinical features on a long-term basis.

## Materials and Methods

### Studied area

**Fig. 1:**
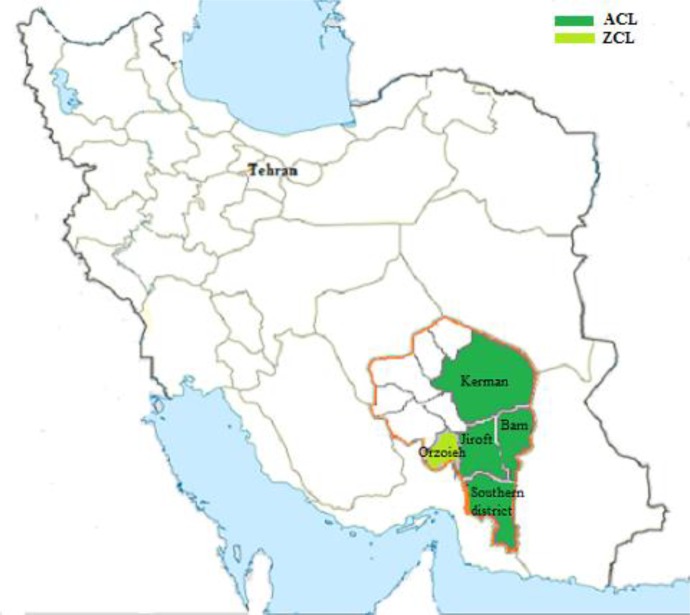
Geographical distribution of anthroponotic cutaneous leishmaniasis (ACL) in districts of Kerman Province, southeastern Iran

Kerman Province with the population of 3 million people covers the area of 181714 km^2^ located 1080 km far from Tehran, southeast Iran (Fig.1). It is the largest province of Iran and covers 11% of total land of the country. *L. tropica* is the only species caused ACL in the endemic areas of Bam (63.6%), Kerman (24.7%), and Jiroft (4.5%) districts, where most of the cases occur and identification of the causative agent and consequently clinical features of the lesions were assessed. The remaining cases (7.2%) occur in other districts of the province (16). Kerman Province has been a well-known target of ACL, in which the field trials of single and multiple doses of *L. major* against *L. tropica* had been conducted (17).

### Data collection

The present study was randomly performed as a descriptive cross-sectional survey in several series between 1994 and 2014. The basis for selecting the cases was identification of the causative agents by intrinsic methods. The representative pictures were prepared in two manners. First, a retrospective survey was designed to assess the samples and related images from the archives of the patients in Bam and Kerman cities (1994–2002) referring to the health clinics for CL treatment where the majority of the CL patients receive proper treatment. Second, prospective surveys in ACL endemic foci were carried out by active and passive case-detection approaches using the health surveillance system (2003–2014).

### Confirmation of cases

Prior to the preparation of the specimens, for each suspected individual, a questionnaire was completed which could record demographic and clinical data. The samples were prepared from the periphery of suspicion active skin lesions, smeared on a glass slide, fixed with methanol, stained by Giemsa, and observed under a light microscope for the presence of amastigotes (Leishman bodies). Since CL imposes a major impact on individuals in Kerman Province, it has long been a noticeable disease and integrated into the health surveillance system. In addition, for nearly 600 cases, at least one or a combination of intrinsic methods from direct smear preparations or a previously cultured organism was used. The identification techniques included PCR-based methods: conventional or nested-PCR, isoenzyme, ELISA, and/or indirect immunofluorescent methods using monoclonal antibodies (16).

Photographs were taken at the first presentation with a lesion. Every lesion on the patient was photographed from the same distance with a case identification number while date was clearly visible in the image to be used for the assessment of clinical forms.

### Nested polymerase chain reaction

DNA preparation and nested PCR were performed according to the method previously used (18). Briefly, this reaction involved two sets of primers: first pair for the outer larger fragment; CSB1XR (ATTTTTCGCGATTTTCGCAGAACG) and CSB2XF (CGAGTAGCAGAAACTCCCGTTCA) and second set for the specific inner fragment: 13Z (ACTGGGGGTTGGTGTAAAATAG) and LiR (TCGCAGAACGCCCC) for the amplification of variable mini-circle fragments of *Leishmania* kinetoplast DNA.

### Ethical issues

The protocols were reviewed and subsequently approved by Ethics Committee of Kerman University of Medical Sciences (project numbers: 91/481 and TDR projects 931190, 980492). Consent of the patients was obtained and the confirmed cases were referred to a local physician for CL treatment. All the patients were treated free of charge using appropriate drugs. An expert dermatologist who had good experience in the field of CL. evaluated clinical forms.

### Data analysis

The statistical analysis was performed using SPSS 20 (Chicago, IL, USA). Chi-square test (*x*^2^) was used for determining statistical differences between demographic data and clinical characteristics of the disease. *P*< 0.05 was considered significant.

## Results

Totally, 2000 randomly selected CL patients (out of 21000 cases) aged 2–96 yr old (mean of 24.6±19.9 yr) who included 897 males (44.8%) and 1103 females (55.2%) were examined clinically for the presence of active lesions (Table 1). Children aged <10 yr old (33.8%), particularly males, were the most infected patients, compared with the other age groups (*P*<0.001).

**Table 1:** Sex and age distribution of anthroponotic cutaneous leishmaniasis patients, Kerman Province, southeastern Iran, 1994–2014

***Age (yr)***	***Male No.* (%)**	***Female No.* (%)**	***Total No.* (%)**
<10	349(38.9)	328(29.7)	677(33.8)
10–20	169(18.9)	166(15.0)	335(16.7)
20–30	130(14.5)	182(16.5)	312(15.7)
30–40	81(9.0)	113(10.3)	194(9.7)
40–50	61(6.8)	111(10.1)	172(8.6)
>50	107(11.9)	203(18.4)	310(15.5)
Total	897(100.0)	1103(100.0)	2000(100.0)

Distribution of ACL lesions on the body in 2000 patients is presented in Table 2. The hands (46.3%) were the most frequent site of involvement, followed by face (34.1%), legs (14.3%), and other parts of the body (5.3%). The number of skin lesions per case ranged from 1 to 9 (mean of 1.5). While most of the patients had single skin lesion (65%), 23% developed two lesions and 12% had three or more lesions. Overall, 1900 (95%) of the patients were indigenous and the remaining cases (5%) were Afghan migrants.

**Table 2:** Location of anthroponotic cutaneous leishmaniasis lesions in patients, Kerman Province, southeastern Iran, 1994–2014

***Location***	***Male No.* (%)**	***Female No.* (%)**	***Total No.* (%)**
Hands	350 (39.1)	575 (52.0)	925 (46.3)
Face	344 (38.4)	339 (30.8)	683 (34.1)
Legs	131 (14.6)	154 (13.9)	285 (14.3)
Others	70 (7.9)	37 (3.3)	107 (5.3)
Total	895 (100.0)	1105 (100.0)	2000(100.0)

Among the typical presentations (Table 3), papule and ulcerated nodule were the most prevalent clinical forms (36.8% and 20.7%, respectively), followed by plaque (18.4%) and ulcerated plaque (18.5%), while among atypical clinical features of the lesions, leishmaniasis recidivans (4.7%) and leishmanid (0.3%) were the dominant forms and followed by diffuse, (0.1%), disseminated (0.1%), sporotrichoid (0.1%), erysipeloid (0.1%), lymphedematous (0.05%), lymphadenic (0.05%), hyperkeratotic (0.05%), paronychial (0.05%), and mutilating (0.05%) types. Altogether, 95.1% of the patients had localized CL lesions at the site of sand fly bites and 4.9% of the cases had chronic cutaneous lesions.

**Table 3:** Clinical features of anthroponotic cutaneous leishmaniasis patients, Kerman Province southeastern Iran, 1994–2014

***Clinical feature***	***No.* (%)**
**Typical forms**	
Papule	736 (36.8)
Plaque	367 (18.4)
Ulcerated nodule	414 (20.7)
Ulcerated plaque	370 (18.5)
**Atypical forms**	
Leishmaniasis recidivans	94 (4.7)
Leishmanid	6 (0.3)
Diffuse cutaneous lesion	2(0.1)
Dissiminated	2 (0.1)
Sporotrichoid	2 (0.1)
Lymphedema	1(0.05)
Lymphadenic	1(0.05)
Hyperkeratotic	1(0.05)
Paronychial	1(0.05)
Multilating	1(0.05)
**Total**	2000(100.0)

**Fig. 2:**
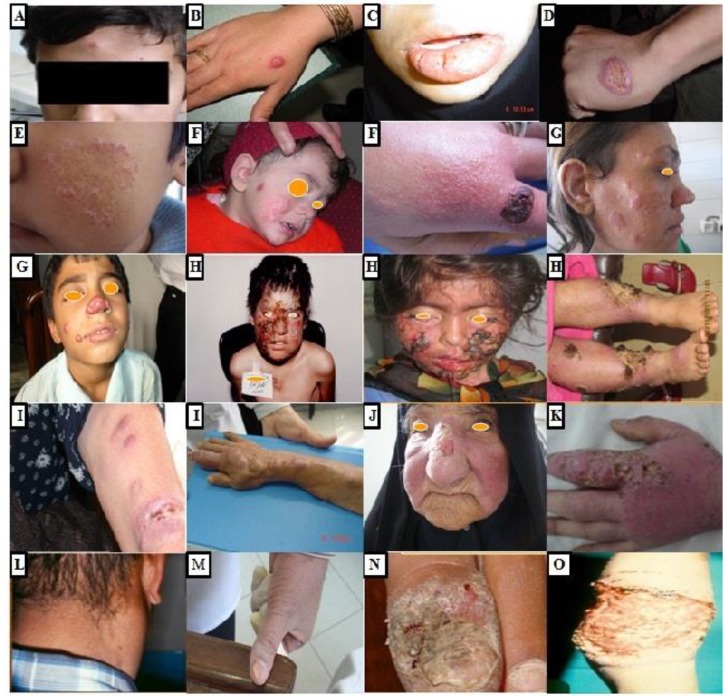
Representative pictures of skin lesions from anthroponotic cutaneous leishmaniasis areas of Kerman Province, southeastern Iran, 1994–2014: **A**. Papule, **B**. Plaque **C**. Ulcerated nodule, **D**. Ulcerated plaque, **E**. Leishmaniasis recidivans, **F**. Leishmanid reaction, **G**. Diffuse cutaneous types, **H**. Dissiminated types, **I**. Sporotrichoid, **J**. Erysipeloid, **K**. lymphedematous type, **L**. Lymphadenic type, **M**. Hyperkeratotic type, **N**. Paronychial type, **O**. Multilating lesion

Representative pictures of the skin lesions from ACL areas of Kerman Province studied here are presented in Fig. 2. Papular lesions, the most prevalent typical form (36.8%), were red, mildly indurated, firm, non-tender, and about 5mm in diameter (Table 3, Fig. 2A). Plaque was red in appearance and covered with scales and follicular hyperkeratosis composed of 18.4% of the lesions (Table 3, Fig. 2B). Ulcerated nodule represented 20.7% of the clinical manifestation. Lesions were usually developed after papule and positioned subcutaneously with firm-elevated, erythematous, and ulcerated surface (Table 3, Fig. 2C). Ulcerated plaque constituted 18.5% of the total clinical forms of the lesions. Such lesions were erythematous and slowly progressed to a painless ulcerated plaque with raised indurated borders (Table 3, Fig. 2D). Leishmaniasis recidivans or lupoid leishmaniasis: Initially, lesions were developed to various stages including papules, nodules, plaques, and scars. The lesions become activated from several months to 2–5 yr after initial improvement. This clinical feature covered 4.7% of the total patients, mostly children in the age group of <10 yr old (Table 3, Fig. 2E). They rarely responded to standard treatment, but a combination of Glucantime along with allopurinol was relatively effective. Leishmanid or id reaction was evident in 0.3% of the CL cases. Leishmanid is presented as sudden, usually symmetrical, eruption in distant sites from primary CL lesion. The most common type of leishmanid reaction was papular form (Table 3, Fig. 2F). Diffuse CL type (0.1%) was characterized by the chronic and progressive spread of non-ulcerative lesions to other parts of the skin. Lesions were most commonly central on the face of two immunosuppressed patients. Treatment with Glucantime was effective in one patient, but it was unsatisfactory in the other (Table 3, Fig. 2G).

Disseminated CL cases (0.1%) were developed following the *L. tropica* lesions in two 5–8 yr old patients, both of whom were immuno-suppressed and eventually died despite extensive efforts to save their lives. This clinical form was characterized by the presence of multiple bizarre shapes and ulcerated lesions, accompanied by visceral and systematic involvement of reticuloendothelial organs by *L. tropica*. The patients were unresponsive to glucantime and amphotericin B (Table 3, Fig. 2H).

Sporotrichoid type lesions were rare in ACL foci and appeared in 0.1% of the total cases. This clinical form presented as multiple non-ulcerative linear nodules across the primary lesion. The nodules are due to lymph node chain involvement (Table 3, Fig. 2I).

Erysipeloid type lesion occurred in 0.1% of the cases, commonly over the nose, usually in women aging 55–60 yr old, which looks like erysipelas clinically. The inflamed lesions were painless and did not ulcerate, contained numerous Leishman bodies, responded well to treatment, and left no prominent scars (Table 3, Fig. 2J).

Lymphedematous type lesion was seen on the left hand of a 65 yr -old woman (0.05%), accompanying local lymphedema with cutaneous lesions. The involvement of lymphatic drainage progressed to the infiltration of lymph and consequently enlargement of the affected organ (Table 3, Fig. 2K).

Lymphadenic type as a rare clinical feature was evident in one case (0.05%). The patient displayed enlarged cervical and submental lymph nodes without any cutaneous involvement. They were approved by excision biopsy of nodes (Table 3, Fig. 2L).

Hyperkeratotic type: Papulonodular or plaques resemble psoriasis covered with dry, horny build dead skin cells. This presentation was observed in one patient (0.05%) (Table 3, Fig. 2M).

Paronychial CL type (0.05%); lesion involved the posterior nail folds with secondary destruction (Table 3, Fig. 2N).

Mutilating type, a rare clinical form (0.05%), started as an erythematous plaque and became larger over few weeks to form an ulcerated nodule. It then was ulcerated and eventually led to the destruction of the skin and underlying tissues (Table 3, Fig. 2O).

**Fig. 3:**
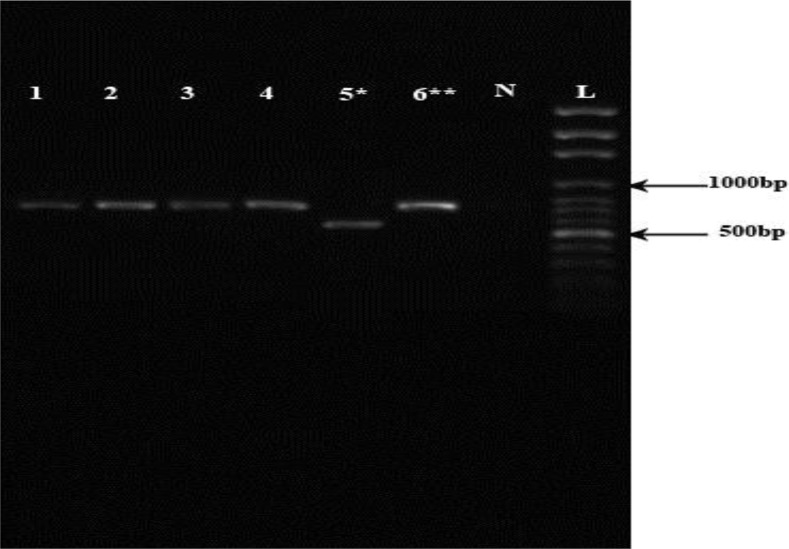
Agarose gel electrophoresis of representative *Leishmania* isolates from anthroponotic cutaneous leishmaniasis areas in Kerman Province, southeastern Iran. Lane L, DNA size marker, 100bp; lane N, negative control (distilled water); lane 6, *L. major* (positive control 570 bp); lane 5, *L. tropica* (positive control 720 bp); lanes1, 2, 3, 4, *L. tropica* (clinical isolates, 720 bp).

In the present study, overall, 600 isolates were identified by various intrinsic methods; 543 specimens’ previously (16), and 57 cases currently by nested PCR (Fig. 3). Based on these analyses, the parasites isolated from the lesions were characterized as *L. tropica*.

## Discussion

CL lesions are present in several clinical forms as indicated in this study. Females were more frequently infected than the corresponding male group, probably related to the fear of cosmetic disfiguration in female cases more than males that urge them to seek medical treatment (19).

ACL occurred in all age groups throughout the year; but, in endemic foci, it was more frequent among children than other older groups. One possible explanation for such an occur-rence might be the outdoor activity of children in sand fly breeding areas, where they contact the CL infection. Moreover, due to the immature development of immune organs, children may be more susceptible. CL cases usually increased with age up to 10–15 yr, after which the prevalence was leveled off. Hands were the more frequent site of involvement in this study; however, there are reports of predominant involvement of face and then hands in this area. After 2003 Bam earthquake, the hands were the most frequent site of involvement in CL patients, as has been already reported in several studies (20).

LR is a rare atypical form of CL lesion found in the areas of endemic for *L. tropica* infection in the old world and manifested usually by facial lesions that resemble lupus vulgaris and may persist for years (14, 21). LR may be difficult to identify in the lesions, as there are no amastigotes (Leishman bodies) in biopsy or tissue smear preparation. Leishmanid or id reaction is a very rare clinical presentation of CL in which eruptions appear in different sites from the primary focus of CL lesion (20, 22). Such eruptions do not contain amastigotes and appear as a part of antigenic cross-reactivity response to the organism or its products.

DCL is also a rare form recognized primarily in East Africa where *L. aethipica* is present (8); but, it occurs rarely by *L. tropica* in ACL endemic areas. A small proportion of it causes DCL type, which does not significantly differ in features from its counterpart in the Old World due to *L. aethiopica*. In this form of CL, nodules may not ulcerate and could persist for two to five years and become chronic (15). Non–ulcerative nodules disseminate from the initial site of infection. DCL type is difficult to treat and is rarely self-healed (11). Multiple lesions form on the skin in association with anergy to leishmaniasis antigens. Defective host defense mechanism in these patients is also a predisposing factor (23).

In contrast, some disseminated types began with a single lesion, eventually ulcerated, progressed, and disseminated to cover the entire body and treatment was ineffective in the present patients. This form is also the result of poor host immune response to the parasite (24). Such presentation was unique since there was no clinical feature reported, previously and huge cutaneous lesions accompanied by involvement of visceral organs, simultaneously.

Erysipeloid form is a rare and unusual manifestation worldwide (range; 0.05%–3.2%). CL cases of Erysipeloid forms have been reported from ZCL foci in Iran (9, 25, 26), Pakistan (27), and Tunisia (28). Similar to this study, most investigators have reported this clinical form over the face covering the nose and sometimes extending onto the cheeks (28). Sporotrichosis CL form is also reported predominantly from ZCL foci (9, 29), but rarely from ACL endemic areas. *L. tropica* has also potential to cause other clinical manifestations (8). Mucocutaneous leishmaniasis (MCL), usually is caused by *L. braziliesis,* similarly, a form of MCL due to *L. tropica* is also reported from Iran (30).

The typical clinical manifestations present no diagnostic difficulties, readily respond to standard treatment, and account for 95.1% of all the clinical forms of ACL, consistent with the previous study in ZCL focus of Isfahan Province (9). In contrast, the lesions with atypical presentations are less frequently manifested because of incomplete course of treatment as well as generation of specific types of immune responses to *Leishmania* infection, species and strains specificity of the causative agent. In fact, due to the complexity of life cycle, presence of wide range of hosts and genetic variability of the *Leishmania* organism, determinants for the clinical polymorphism are still unknown (11). *L. tropica* is very heterogeneous as demonstrated by different immunological, biochemical, and molecular techniques (12). However, two different *L. tropica* strains (MON-39 and MON-55) have been previously reported (16). This species is also associated with a broad and complex clinical spectrum of ACL represented by numerous cases of simple CL and diverse uncommon clinical presentations (8). The main limitation for performing the present study was the selection of those cutaneous cases identified to the species level. Although the area is major for ACL cases, there might be a limited number of strain variability that could, in turn, restrict diversity of the clinical forms.

## Conclusion

ACL is associated with a broad and complex spectrum of clinical features, which mimic other disease conditions. The disease represents numerous cases of localized form and diverse uncommon clinical presentations. Atypical clinical forms of CL occur because of various factors, mainly host immunology, incomplete treatment, and genetic variants of the causative agent. Such forms are predominantly unresponsive to standard treatment and may persist for a long period, play a significant role in the morbidity of the disease, and lead to destructive and disfiguring consequences. Hence, physicians should be aware of such diverse presentations, particularly in endemic areas. Hence, precise identification of the causative agent and effective treatment modality should have high priority for improving control strategies, particularly in ACL endemic foci.
